# The analysis of the lipid levels in patients with coronary artery disease after percutaneous coronary intervention: a one-year follow-up observational study

**DOI:** 10.1186/s12944-020-01340-5

**Published:** 2020-07-06

**Authors:** Weiyu Qiu, Jiali Chen, Xianzhen Huang, Jun Guo

**Affiliations:** 1 Department of Cardiology, The First Affiliated Hospital of Jinan University, 613 Huangpu Da Dao Xi, Guangzhou, 510630 Guangdong China; 2 Department of Transitional care, The First Affiliated Hospital of Jinan University, 613 Huangpu Da Dao Xi, Guangzhou, 510630 Guangdong China

**Keywords:** Serum lipids, Percutaneous coronary intervention, Prevalence, Risk factors, Follow-up, Observational study

## Abstract

**Background:**

Coronary heart disease (CHD) is one of the leading causes of death worldwide. Percutaneous coronary intervention (PCI) has been an important technology for the treatment of CHD. Blood lipid management is critical for PCI patients because not only should local vascular pathological changes be considered but the whole atherosclerotic process should be considered as well.

**Methods:**

A total of 522 patients diagnosed with CHD (including acute myocardial infarction and unstable angina) successfully underwent stent implantation in acute or elective PCI in the cardiology department of one general hospital in Guangzhou from June 2015 to December 2017. The 2016 Chinese Guideline for the Management of dyslipidaemia in Adults and the National Cholesterol Education Program (NCEP) Expert Panel on Detection, Evaluation, and Treatment of High Blood Cholesterol in Adults (Adult Treatment Panel III) final report (NCEP-ATP III) were used to classify total cholesterol (TC), triglyceride (TG), low-density lipoprotein cholesterol (LDL-C) and high-density lipoprotein cholesterol (HDL-C) levels.

**Results:**

A total of 522 patients were recruited for the study. The mean values of TC, TG, LDL-C, and HDL-C at baseline were 4.76, 1.80, 2.93 and 1.03 mmol/L, respectively. After 1 year of follow-up, the mean values of TC, TG, LDL-C, and HDL-C were 3.94, 1.62, 2.26 and 1.01 mmol/L, respectively. The prevalence of high TC, high TG, high LDL-C and low HDL-C at baseline was 12.05, 21.80, 10.90 and 56.79%, respectively, and the prevalence at follow-up was 4.59, 15.68, 3.25 and 59.85%, respectively. Logistic regression revealed that gender was risk factor for high TC (≥ 6.22 mmol/L), low HDL-C (< 1.04 mmol/L) and high LDL-C (≥ 4.14 mmol/L) at follow-up. Age was the factor associated with high TG (≥ 2.26 mmol/L) and low HDL-C (< 1.04 mmol/L) at follow-up. Besides, smoking and diet control were risk factors for low HDL-C (< 1.04 mmol/L) and high LDL-C (≥ 4.14 mmol/L) at follow-up, respectively.

**Conclusion:**

The patients with PCI at follow-up experienced lower mean values of lipids and prevalence of dyslipidaemia than those at baseline. Gender, age, smoking and diet control were the risk factors associated with elevated lipids. Improvement in lipid management at follow up demonstrated that such intervention can be effective.

## Introduction

Due to urbanization, economic growth and an ageing population, China’s disease spectrum has changed. Coronary heart disease (CHD) has become a public health issue and is the leading cause of death globally [1, 2]. CHD is not only an important cause of death in developed countries but also in developing countries [3]. One of the treatment strategies for CHD is to relieve severe coronary occlusion, and another strategy is to control risk factors. Although percutaneous coronary intervention (PCI) has been an important technology for the treatment of CHD, it can only cause local vascular pathological changes [4], and the entire atherosclerotic process should be considered. Therefore, postoperative management after PCI is essential.

Abnormal blood lipid metabolism is a risk factor for CHD [5]. The increase in total cholesterol (TC) and low-density lipoprotein cholesterol (LDL-C) and the decrease in high-density lipoprotein cholesterol (HDL-C) levels are the main factors associated with cardiovascular disease [6]. Therefore, blood lipid levels play a crucial role in the development of CHD. Previous studies have shown that TC, LDL-C and triglyceride (TG) levels were increasing [7, 8], and hypercholesterolemia was not adequately controlled in Chinese adults aged 35 to 74 years [7]. Meta-analysis results showed that an LDL-C reduction of 1 mmol/L can effectively reduce the incidence of cardiovascular events [9].

Previous studies on dyslipidaemia have been conducted in individuals with chronic diseases such as hypertension and diabetes [10], obese people [11] and the Chinese general population [7, 12–14]. There are relatively few studies on the management of dyslipidaemia in patients with PCI, and there are even fewer follow-up studies. The following hypothesis was proposed: Follow-up is helpful to control the lipid profiles in PCI patients. Therefore, this study aimed to explore the blood lipid status of patients after PCI through a one-year follow-up period.

## Materials and methods

### Subjects

This retrospective analysis of 522 patients diagnosed with CHD (including acute myocardial infarction and unstable angina) successfully underwent stent implantation in acute or elective PCI in the cardiology department of one general hospital in Guangzhou from June 2015 to December 2017.

The sample size estimation was calculated using the following formula: $n={\left[{z}_{\alpha}\sqrt{2\overline{p}\overline{q}}+{z}_{\beta}\sqrt{p_0{q}_0+{p}_1{q}_1}\right]}^2/{\left({p}_1-{p}_0\right)}^2$, where Zα = 1.96; Z_β_ = 1.282; p_0_ = 0.081; q_0_ = 1 - p_0_ = 0.919; p_1_ = RR * p_0_ = 2.5 * 0.081 = 0.203; q_1_ = 1 – p_1_ = 0.797; $\overline{p}$ =p_0_ + p_1_/2 = 0.142; and $\overline{q}$ =1 - $\overline{p}$ =0.858. According to the formula, the sample size was estimated to be 169 subjects with a 10% loss to follow-up; therefore, the number of patients included in the study was 186.

### Study procedure

This study was conducted by a case manager. Because it is typical for patients who undergo PCI to stay in the cardiac intensive care unit (CCU) for one night, health education (focused on medication guidance, lifestyle, and follow up) for patients was first conducted by CCU nurses, and then the next day upon transfer to the general ward, the nurses in the general ward continued providing education to the patients. The case manager registered the patients’ information and created a follow-up form. For the patients included in 2015 and 2016, the case manager followed up the patients discharged from the hospital after 1 year by telephone, gathering data including information on chest pain, medicine, follow-up, lifestyle, cardiac function. For patients in 2017, telephone follow-up was performed at 1 month, 3 months, 6 months and 1 year after discharge, and data were collected including information on medication, discomfort, complications, follow-up, lipids, colour Doppler sonography results and lifestyle. Patients in 2015 and 2016 were verbally informed about the follow-up procedure. Patients in 2017 were informed about a follow-up plan after which they provided written informed consent. To control the bias, the case manager was responsible for the whole process of recording the information in the database and the follow-up plan.

### Study parameters

The data were collected from medical records and face-to-face or telephone interviews. The information included demographic characteristics (name, gender, age, ethnicity, telephone number, residence, education, marital status, occupation, family monthly income), medical history (hypertension, diabetes, CHD, stroke and dyslipidaemia), lifestyle and self-management (diet control, daily staple food intake, regular exercise, weekly exercise time, smoking and drinking history), physical examination (height, weight), and laboratory examination (blood lipids at baseline and follow-up). The lipids were obtained in the morning after an overnight fast. TC (enzymatic method), TG (enzymatic method), HDL-C (direct method), and LDL-C (direct method) levels were measured.

### Definition

The 2016 Chinese Guidelines for the Management of Dyslipidaemia in Adults were used to classify TC, TG, LDL-C and HDL-C levels [15]. The classification was based on the criteria in the Third Report of the National Cholesterol Education Program (NCEP) Expert Panel on Detection, Evaluation, and Treatment of High Blood Cholesterol in Adults (Adult Treatment Panel III) final report (NCEP-ATP III) [16]. The diagnostic criteria of diabetes mellitus (DM) were based on the 1999 WHO diagnostic criteria [17]. DM was defined as fasting plasma glucose (FPG) ≥ 7.0 mmol/L, 2-h postprandial plasma glucose (2hPG) ≥ 11.1 mmol/L, or diabetic symptoms along with a random plasma glucose level ≥ 11.1 mmol/L. Hypertension was defined as systolic blood pressure (SBP) ≥ 140 mmHg and/or diastolic blood pressure (DBP) ≥ 90 mmHg. High TC was defined as TC ≥ 6.22 mmol/L. High LDL-C was defined as LDL-C ≥ 4.14 mmol/L, low HDL-C was defined as HDL-C < 1.04 mmol/L, and high TG were defined as ≥2.26 mmol/L. Based on the status of self-reported smoking and the definition of the global adult tobacco survey [18], individuals were categorized into three groups: never, former and current smokers. Respondents were asked whether they had a drink over the past year. According to the adult weight standard published by the Ministry of Health of China, body mass index (BMI) < 18.5 kg/m^2^ indicated low weight, 18.5 kg/m^2^ ≤ BMI < 24 kg/m^2^ indicated normal weight, 24 kg/m^2^ ≤ BMI < 28 kg/m^2^ indicated overweight and BMI ≥ 28 kg/m^2^ indicated obesity. Light physical activity referred to sitting, standing or walking, and activities not requiring special muscular function, such as reading, writing, performing office work, assembling and repairing machines, lecturing, performing general laboratory operations, doing housework. Moderate physical activity referred to activities with greater muscular requirements, such as performing daily activities of students, driving motor vehicles, installing electrical components, cutting metal, performing woodworking operations. Heavy physical activity referred to nonmechanized agricultural labour, steelmaking, lathe operation, sports activities (swimming, mountain climbing, football). Regular exercise was defined as exercising at least 3 times a week for at least 30 mins at a time.

### Data analysis

All data were analysed using SPSS version 13.0 software. Quantitative data were expressed as the means ± SD. Categorical variables were estimated as frequencies. The comparisons of quantitative data were conducted using the independent samples *t*-test, and categorical variables were compared using the chi-square test. The comparison between lipid profiles at baseline and follow-up was performed using a paired t test. Binary logistic regression analysis was used to assess the risk factors. Odds ratios (ORs) with 95% confidence intervals (95%CIs) were expressed. *P* < 0.05 was considered statistically significant.

## Results

### Demographic and clinical features of patients

**Fig. 1:**
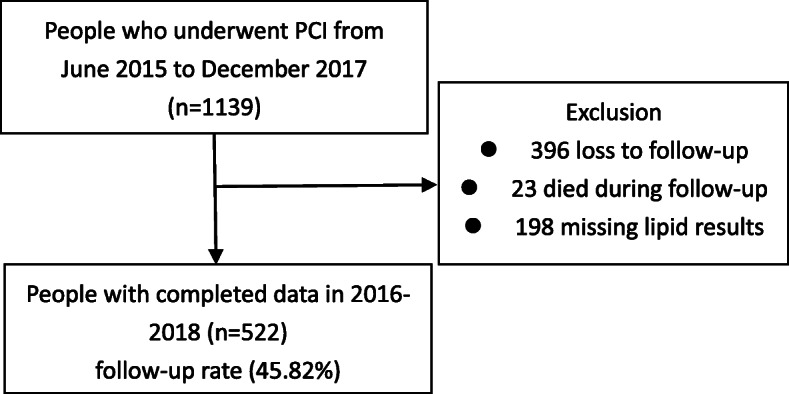
Flow diagram of patients analysed in the study

Figure 1 shows the flowchart of participants analysed in the study. A total of 522 patients aged 28–88 years (63.15 ± 11.38 years), including 393 males (75.3%) and 129 females (24.7%), were enrolled in the study. According to the LDL-C levels at baseline, patients were divided into five groups: < 2.59 mmol/L, 2.59 mmol/L − < 3.37 mmol/L, 3.37 mmol/L − < 4.14 mmol/L, 4.14 mmol/L − < 4.92 mmol/L, and ≥ 4.92 mmol/L. The differences in age, gender, BMI, education, residence, smoking, alcohol consumption, occupation, family monthly income, diet control, daily staple food intake and physical activity were not significant (*P* > 0.05) (Table 1). In addition, patients were divided into three groups separately based on TC, TG and HDL-C levels at baseline. The differences in TG levels at baseline according to age, BMI, education, drinking and daily staple food intake were significant (*P* < 0.05). Gender, BMI, smoking and alcohol consumption were significantly associated with HDL-C levels at baseline (*P* < 0.05) (Table 2).

**Table 1:** Characteristics of the patients according to the LDL levels at baseline

Characteristics	LDL-C levels						
	< 2.59	2.59- < 3.37	3.37- < 4.14	4.14- < 4.92	≥4.92	χ^2^	*P*-value
Age (years), n(%)						29.793	0.073
28–29	0 (0.0)	0 (0.0)	1 (100.0)	0 (0.0)	0 (0.0)		
30–39	5 (38.5)	4 (30.8)	2 (15.4)	2 (15.4)	0 (0.0)		
40–49	14 (29.2)	9 (18.8)	18 (37.5)	5 (10.4)	2 (4.2)		
50–59	41 (30.4)	42 (31.1)	39 (28.9)	9 (6.7)	4 (3.0)		
60–69	71 (44.7)	39 (24.5)	31 (19.5)	16 (10.1)	2 (1.3)		
≥ 70(%)	76 (45.8)	48 (28.9)	25 (15.1)	12 (7.2)	5 (3.0)		
Gender, n(%)						0.637	0.959
Male	156 (39.7)	108 (27.5)	88 (22.4)	31 (7.9)	10 (2.5)		
Female	51 (39.5)	34 (26.4)	28 (21.7)	13 (10.1)	3 (2.3)		
BMI (kg/m^2^), n(%)						11.543	0.483
< 18.5	4 (36.4)	3 (27.3)	3 (27.3)	0 (0.0)	1 (9.1)		
18.5- < 24.0	110 (41.5)	75 (28.3)	53 (20.0)	23 (8.7)	4 (1.5)		
24.0- < 28.0	68 (38.0)	50 (27.9)	38 (21.2)	16 (8.9)	7 (3.9)		
≥ 28.0	25 (37.3)	14 (20.9)	22 (32.8)	5 (7.9)	1 (1.5)		
Education, n(%)						5.006	0.958
Primary school	32 (42.7)	19 (25.3)	15 (20.0)	7 (9.3)	2 (2.7)		
Middle school	82 (38.5)	58 (27.2)	51 (23.9)	16 (7.5)	6 (2.8)		
High school	81 (40.1)	53 (26.2)	43 (21.3)	20 (9.9)	5 (2.5)		
> High school	12 (37.5)	12 (37.5)	7 (21.9)	1 (3.1)	0 (0.0)		
Residence, n(%)						0.267	0.992
Urban	132 (40.4)	88 (26.9)	71 (21.7)	28 (8.6)	8 (2.4)		
Rural	75 (38.5)	54 (27.7)	45 (23.1)	16 (8.2)	5 (2.6)		
Smoking, n(%)						12.097	0.147
Never	131 (43.2)	78 (25.7)	65 (21.5)	22 (7.3)	7 (2.3)		
Former	22 (50.0)	11 (25.0)	6 (13.6)	3 (6.8)	2 (4.5)		
Current	54 (31.0)	53 (30.5)	45 (25.9)	19 (10.9)	3 (1.7)		
Drinking, n(%)						4.553	0.336
Yes	23 (39.0)	11 (18.6)	16 (27.1)	6 (10.2)	3 (5.1)		
No	184 (39.7)	131 (28.3)	100 (21.6)	38 (8.2)	10 (2.2)		
Occupation, n(%)						12.719	0.122
Light	179 (41.7)	115 (26.8)	93 (21.7)	31 (7.2)	11 (2.6)		
Medium	27 (30.3)	24 (27.0)	23 (25.8)	13 (14.6)	2 (2.2)		
others	1 (25.0)	3 (75.0)	0 (0.0)	0 (0.0)	0 (0.0)		
Famliy monthly income per capita, n(%)						9.987	0.617
< 1000	0 (0.0)	0 (0.0)	1 (100.0)	0 (0.0)	0 (0.0)		
1000–3000	58 (37.2)	41 (26.3)	40 (25.6)	13 (8.3)	4 (2.6)		
3000–6000	135 (41.7)	85 (26.2)	68 (21.0)	29 (9.0)	7 (2.2)		
> 6000	14 (34.1)	16 (39.0)	7 (17.1)	2 (4.9)	2 (4.9)		
Diet control, n(%)						5.948	0.203
No	39 (31.7)	33 (26.8)	34 (27.6)	13 (10.6)	4 (3.3)		
Yes	168 (42.1)	109 (27.3)	82 (20.6)	31 (7.8)	9 (2.3)		
Daily staple food intake, n(%)						12.308	0.138
Rice	185 (38.8)	128 (26.8)	112 (23.5)	40 (8.4)	12 (2.5)		
Noodle	1 (14.3)	3 (42.9)	1 (14.3)	2 (28.6)	0 (0.0)		
Rice+Noodle	21 (55.3)	11 (28.9)	3 (7.9)	2 (5.3)	1 (2.6)		
Physical activity, n(%)						6.349	0.175
No	5 (20.0)	8 (32.0)	8 (32.0)	4 (16.0)	0 (0.0)		
Yes	202 (40.6)	134 (27.0)	108 (21.7)	40 (8.0)	13 (2.6)		

*LDL-C* low-density lipoprotein cholesterol, *BMI* body mass index

**Table 2:** Characteristics of the patients according to the other lipid profiles levels at baseline

Characteristics	TC levels			χ^2^	*P*	TG levels			χ^2^	*P*	HDL-C levels			χ^2^	*P*
	< 5.18	5.18- < 6.22	≥6.22			< 1.69	1.69- < 2.26	≥2.26			< 1.04	1.04- < 1.55	≥1.55		
Age (years), n(%)				10.666	0.384				27.759	0.002				12.450	0.256
28–29	1 (100)	0 (0.0)	0 (0.0)			0(0.0)	0(0.0)	1(100.0)			1(100.0)	0(0.0)	0(0.0)		
30–39	7 (53.8)	4 (30.8)	2 (15.4)			3(23.1)	3(23.1)	7(53.8)			9(69.2)	4(30.8)	0(0.0)		
40–49	25 (52.1)	16 (33.3)	7 (14.6)			25(52.1)	8(16.7)	15(31.1)			29(60.4)	18(37.5)	1(2.1)		
50–59	84 (62.2)	39 (28.9)	12 (8.9)			71(52.6)	26(19.3)	38(28.1)			87(64.4)	43(31.9)	5(3.7)		
60–69	107(67.3)	32 (20.1)	20 (12.6)			100(62.9)	29(18.2)	30(18.9)			92(57.9)	61(38.4)	6(3.8)		
≥ 70	113(68.1)	31 (18.7)	22 (13.3)			116(69.9)	27(16.3)	23(13.9)			79(47.6)	76(45.8)	11(6.6)		
Gender, n(%)				5.239	0.073				0.073	0.964				20.184	< 0.001
Male	264(67.2)	87(22.1)	42(10.7)			238(60.6)	69(17.6)	86(21.9)			244(62.1)	137(34.9)	12(3.1)		
Female	73 (56.6)	35(27.1)	21(16.3)			77(59.7)	24(18.6)	28(21.7)			53(41.1)	65(50.4)	11(8.5)		
BMI (kg/m^2^), n(%)				1.748	0.941				19.792	0.003				22.786	0.001
< 18.5	7(63.6)	3(27.3)	1(9.1)			10(90.9)	0(0.0)	1(9.1)			2(18.2)	6(54.5)	3(27.3)		
18.5- < 24.0	176(66.4)	61(23.0)	28(10.6)			171(64.5)	51(19.2)	43(16.2)			152(57.4)	104(39.2)	9(3.4)		
24.0- < 28.0	113(63.1)	41(22.9)	25(14.0)			104(58.1)	29(26.2)	46(25.7)			112(62.6)	60(33.5)	7(3.9)		
≥ 28.0	41(61.2)	17(25.4)	9(13.4)			30(44.8)	13(19.4)	24(35.8)			31(46.3)	32(47.8)	4(6.0)		
Education, n(%)				5.451	0.487				13.340	0.038				9.685	0.139
Primary school	46(61.3)	17(22.7)	12(16.0)			56(74.7)	7(9.3)	12(16.0)			31(41.3)	39(52.0)	5(6.7)		
Middle school	134(62.9)	55(25.8)	24(11.3)			128(60.1)	39(18.3)	46(21.6)			124(58.2)	80(37.6)	9(4.2)		
High school	135(66.8)	41(20.3)	26(12.9)			110(54.5)	39(19.3)	53(26.2)			124(61.4)	71(35.1)	7(3.5)		
> High school	22(68.8)	9(28.1)	1(3.1)			21(65.6)	8(25.0)	3(9.4)			18(56.3)	12(37.5)	2(6.3)		
Residence, n(%)				4.407	0.354				5.584	0.232				0.945	0.918
Urban	211(64.5)	73(22.3)	43(13.1)			200(61.2)	62(19.0)	65(19.9)			184(56.3)	129(39.4)	14(4.3)		
Rural	126(64.9)	48(24.7)	20(10.3)			115(59.3)	31(16.0)	48(24.7)			112(57.7)	73(37.6)	9(4.6)		
Smoking, n(%)				0.459	0.977				7.117	0.130				25.007	< 0.001
Never	196(64.7)	70(23.1)	37(12.2)			195(64.4)	50(16.5)	58(19.1)			148(48.8)	142(46.9)	13(4.3)		
Former	27(61.4)	12(27.3)	5(11.4)			28(63.6)	7(15.9)	9(20.5)			26(59.1)	14(31.8)	4(9.1)		
Current	114(65.5)	40(23.0)	20(11.5)			91(52.3)	36(20.7)	47(27.0)			123(70.7)	46(26.4)	5(2.9)		
Drinking, n(%)				0.174	0.917				7.388	0.025				8.011	0.018
Yes	38(64.4)	13(22.0)	8(13.6)			29(49.2)	9(15.3)	21(35.6)			42(71.2)	13(22.0)	4(6.8)		
No	299(64.6)	109(23.5)	55,911.9)			286(61.8)	84(18.1)	91(20.1)			255(55.1)	189(40.8)	19(4.1)		
Occupation, n(%)				3.814	0.432				1.908	0.753				1.772	0.778
Light	284(66.2)	95(22.1)	50(11.7)			257(59.9)	79(18.4)	93(21.7)			245(57.1)	163(38.0)	21(4.9)		
Medium	50(56.2)	26(29.2)	13(14.6)			55(61.8)	13(14.6)	21(23.6)			50(56.2)	37(41.6)	2(2.2)		
others	3(15.0)	1(25.0)	0(0.0)			3(75.0)	1(25.0)	0(0.0)			2(50.0)	2(50.0)	0(0.0)		
Famliy monthly income per capita, n(%)				8.757	0.188				10.815	0.094				5.359	0.499
< 1000	0(0.0)	1(100.0)	0(0.0)			1(100.0)	0(0.0)	0(0.0)			0(0.0)	1(100.0)	0(0.0)		
1000–3000	98(62.8)	44(28.2)	14(9.0)			99(63.5)	20((12.8)	37(23.7)			86(55.1)	60(38.5)	10(6.4)		
3000–6000	213(65.7)	66(20.4)	45(13.9)			191(59.0)	60(18.5)	73(22.5)			189(58.3)	125(38.6)	10(3.1)		
> 6000	26(63.4)	11(26.8)	4(9.8)			24(58.5)	13(31.7)	4(9.8)			22(53.7)	16(39.0)	3(7.3)		
Diet control, n(%)				5.086	0.079				1.650	0.438				4.976	0.083
No	69(56.1)	35(28.5)	19(15.4)			70(56.9)	21(17.1)	32(26.0)			60(48.8)	55(44.7)	8(6.5)		
Yes	268(67.2)	87(21.8)	44(11.0)			245(61.4)	72(18.0)	82(20.6)			237(59.4)	147(36.8)	15(3.8)		
Daily staple food intake, n(%)				3.645	0.456				9.822	0.044				8.838	0.065
Rice	306(64.2)	113(23.7)	58(12.2)			281(58.9)	90(18.9)	106(22.2)			280(58.7)	178(37.3)	19(4.0)		
Noodle	3(42.9)	2(28.6)	2(28.6)			3(42.9)	1(14.3)	3(42.9)			2(28.6)	4(57.1)	1(14.3)		
Rice + Noodle	28(73.7)	7(18.4)	3(7.9)			31(81.6)	2(5.3)	5(13.2)			15(39.5)	20(52.6)	3(7.9)		
Physical activity, n(%)				1.581	0.454				0.209	0.901				0.025	0.988
No	15(60.0)	5(20.0)	5(20.0)			14(56.0)	5(20.0)	6(24.0)			14(56.0)	10(40.0)	1(4.0)		
Yes	322(64.8)	117(23.5)	58(11.7)			301(60.6)	88(17.7)	108(21.7)			283(56.9)	192(38.6)	22(4.4		

*TC* total cholesterol, *TG* triglyceride, *LDL-C* low-density lipoprotein cholesterol, *HDL-C* high-density lipoprotein cholesterol, *BMI* body mass index

The mean values of TC, TG, LDL-C, and HDL-C at baseline were 4.76, 1.80, 2.93 and 1.03 mmol/L, respectively. After 1 year of follow-up, the mean TC, TG, LDL-C, and HDL-C levels were 3.94, 1.62, 2.26 and 1.01 mmol/L, respectively. There was a significant difference in TC, TG and LDL-C levels between baseline and follow-up (*P* < 0.05) (Table 3). The prevalence of high TC, high TG, high LDL-C and low HDL-C at baseline was 12.05, 21.80, 10.90 and 56.79%, respectively, and the prevalence at follow-up was 4.59, 15.68, 3.25 and 59.85%, respectively. The difference in prevalence between baseline and follow-up was significant (*P* < 0.05) (Table 4).

**Table 3:** The comparison of lipid profiles between baseline and follow-up

Characteristics	At baseline	At follow-up	t	*P*
TC	4.76 ± 1.25	3.94 ± 1.05	11.456	< 0.001
TG	1.80 ± 1.31	1.62 ± 1.25	2.256	0.024
LDL-C	2.93 ± 0.94	2.26 ± 0.81	12.428	< 0.001
HDL-C	1.03 ± 0.28	1.01 ± 0.28	1.082	0.279

*TC* total cholesterol, *TG* triglyceride, *LDL-C* low-density lipoprotein cholesterol, *HDL-C* high-density lipoprotein cholesterol

**Table 4:** The comparison of high lipid prevalence between baseline and follow-up

Characteristics	Prevalence (%)		χ^2^	*P*
	At baseline	At follow-up		
High TC	12.05	4.59	19.072	< 0.001
High TG	21.80	15.68	6.432	0.011
High LDL-C	10.90	3.25	23.271	< 0.001
Low HDL-C	56.79	59.85	1.220	0.269

*TC* total cholesterol, *TG* triglyceride, *LDL-C* low-density lipoprotein cholesterol, *HDL-C* high-density lipoprotein cholesterol

Among 522 patients included in the 1 year follow-up, a total of 484 patients’ stents were unobstructed, 1 had reimplantation via PCI, 7 had intimal hyperplasia, 1 died, 7 had stent stenosis, 22 were followed up for blood lipids in the outpatient department, and no patients were hospitalized for vascular examination.

As shown in Table 5, the proportion of patients at follow-up with borderline high (5.18 − < 6.22 mmol/L) and high TC levels (≥ 6.22 mmol/L) was 7.1 and 4.6%, respectively, which was higher among those living in rural areas than urban areas. The proportion of patients at follow-up with borderline high (1.69 − < 2.26 mmol/L) and high (≥2.26 mmol/L) TG was 15.7 and 15.7%, respectively, and the proportion was higher in younger patients than in older patients. The proportion of patients at follow-up with low HDL-C was 60.2%, which was much higher in younger patients than in older patients and was higher in men than in women. The proportion of patients at follow-up with borderline high (3.37 − < 4.14 mmol/L), high (4.14 − < 4.92 mmol/L), and very high (≥ 4.92 mmol/L) LDL-C levels was 6.1, 2.1 and 1.1%, respectively. The proportion of patients living in rural areas with borderline high, high and very high LDL-C was significantly higher than that of patients living in urban areas.

**Table 5:** Proportion of lipid fraction serum levels at follow-up according to Chinese guidelines classification

	Overall	Age				Gender			Residence		
		28–49	50–69	≥70	*P*-value	Men	Women	*P*-value	Urban	Rural	*P*-value
TC, mmol/L					0.272			0.136			0.063
Desirable< 5.18	88.3	87.1	87.4	90.4		89.1	86.0		90.5	84.6	
Borderline high5.18- < 6.22	7.1	4.8	8.8	4.8		7.4	6.2		6.4	8.2	
High≥6.22	4.6	8.1	3.7	4.8		3.6	7.8		3.1	7.2	
TG, mmol/L					< 0.001			0.816			0.235
Desirable< 1.69	68.6	46.8	68.0	77.7		68.2	69.8		71.3	64.1	
Borderline high1.69- < 2.26	15.7	21.0	17.7	10.2		16.3	14.0		14.4	17.9	
High≥2.26	15.7	32.3	14,3	12.0		15.5	16.3		14.4	17.9	
HDL-C, mmol/L					0.007			< 0.001			0.857
Low< 1.04	60.2	79.0	59.2	54.8		66.7	40.3		60.6	59.5	
Desirable1.04- < 1.55	35.2	21.0	36.7	38.0		30.0	51.2		34.6	36.4	
High≥1.55	4.6	0.0	4.1	7.2		3.3	8.5		4.9	4.1	
LDL-C, mmol/L					0.254			0.183			0.021
Desirable< 2.59	73.4	62.9	74.1	75.9		74.3	70.5		75.8	69.2	
Near or above optimal2.59- < 3.37	17.2	27.4	16.0	15.7		16.8	18.6		16.5	18.5	
Borderline high3.37- < 4.14	6.1	4.8	6.1	6.6		6.4	5.4		5.8	6.7	
High4.14- < 4.92	2.1	1.6	2.4	1.8		2.0	2.3		1.8	2.6	
Very high≥4,92	1.1	3.2	1.4	0.0		0.5	3.1		0.0	3.1	

*TC* total cholesterol, *TG* triglyceride, *LDL-C* low-density lipoprotein cholesterol, *HDL-C* high-density lipoprotein cholesterol

Multivariate logistic regression analysis showed that gender was risk factor for high TC (≥ 6.22 mmol/L), low HDL-C (< 1.04 mmol/L) and high LDL-C (≥ 4.14 mmol/L) at follow-up. Age was the factor associated with high TG (≥ 2.26 mmol/L) and low HDL-C (< 1.04 mmol/L) at follow-up. Besides, smoking and diet control were risk factors for low HDL-C (< 1.04 mmol/L) and high LDL-C (≥ 4.14 mmol/L) at follow-up, respectively (Table 6).

**Table 6:** The association between factors at baseline and dyslipidemia at follow-up in a binary logistic regression analyses

	TC ≥ 6.22 mmol/L			TG ≥ 2.26 mmol/L			HDL-C < 1.04 mmol/L			LDL-C ≥ 4.14 mmol/L		
	OR	95%*CI*	*P* value	OR	95%*CI*	*P* value	OR	95%*CI*	*P* value	OR	95%*CI*	*P* value
Gender	0.268	0.082,0.875	0.029	0.915	0.471,1.777	0.793	2.754	1.679,4.517	< 0.001	0.153	0.034,0.687	0.014
Age	1.008	0.964,1.054	0.734	1.030	1.005,1.055	0.020	1.025	1.005,1.045	0.014	1.030	0.978,1.086	0.261
BMI	1.011	0.868,1.177	0.889	1.005	0.921,1.097	0.906	1.035	0.968,1.107	0.318	1.196	0.989,1.446	0.064
Education	1.042	0.485,2.236	0.917	1.032	0.670,1.589	0.886	1.376	0.994,1.906	0.054	0.594	0.238,1.484	0.265
Residence	0.522	0.195,1.399	0.196	0.814	0.464,1.427	0.472	1.197	0.766,1.870	0.429	0.352	0.107,1.156	0.085
Smoking	0.690	0.387,1.229	0.208	1.154	0.857,1.554	0.346	0.790	0.626,0.998	0.048	0.806	0.389,1.672	0.563
Drinking	1.627	0.464,5.702	0.447	1.622	0.784,3.355	0.192	0.774	0.416,1.441	0.419	3.643	0.945,14.039	0.060
Occupation	1.115	0.373,3.328	0.845	1.469	0.740,2.917	0.272	0.921	0.587,1.446	0.721	1.260	0.348,4.564	0.725
Famliy monthly income per capita	1.211	0.474,3.090	0.689	1.166	0.680,1.999	0.578	0.757	0.497,1.151	0.193	1.009	0.347,2.935	0.987
Diet control	1.489	0.506,4.382	0.470	1.277	0.667,2.447	0.460	1.048	0.627,1.752	0.857	4.464	1.265,15.750	0.020
Daily staple food intake	2.341	0.514,10.669	0.272	1.329	0.715,2.469	0.368	0.987	0.669,1.454	0.946	/	/	/
Physical activity	1.694	0.328,8.758	0.530	0.737	0.205,2.651	0.640	1.935	0.728,5.139	0.186	2.994	0.508,17.639	0.226

*TC* total cholesterol, *TG* triglyceride, *LDL-C* low-density lipoprotein cholesterol, *HDL-C* high-density lipoprotein cholesterol, *BMI* body mass index

## Discussion

Blood lipid management after PCI is essential because dyslipidaemia is an important risk factor for CHD. This study was performed by recruiting 522 patients from 2015 to 2017 who had undergone PCI. The results showed that a significant decrease in the mean TC, TG and LDL-C levels was observed between baseline and follow-up. Similarly, a significant decrease in the prevalence of high TC, high TG and high LDL-C was observed over the same period. The mean TC, TG, LDL-C, and HDL-C levels at baseline were 4.76, 1.80, 2.93, and 1.03 mmol/L, respectively, which were higher than the mean values of lipids in Chinese adults reported in the 1983, 1993 [13], 2013–2014 [12], and 2002 China National Nutrition and Health Survey [14], respectively. This may be related to the information on myocardial infarction reported in this study. The TC and LDL-C levels were significantly lower in this study than the levels in the United States [19] and Japan [20] but similar to those in Korea [21]. After 1 year of follow-up, the patients’ TC, TG, LDL-C and HDL-C levels were significantly lower than those at baseline, which indicates that post-discharge follow-up management is essential.

The current study confirmed that the prevalence rates of high TC, high TG, high LDL-C and low HDL-C at baseline were 12.05, 21.80, 10.90, and 56.79%, respectively, which were higher than those reported in 2002 [14] and 2013–2014 [12]. This was mainly due to the different populations involved and the different definitions of the indicators. Dyslipidaemia in this population is mainly reflected by lower HDL-C and higher TG, which is consistent with findings in previous studies [12] but different from those in the United States (high TC and high LDL-C) [19], which may be due to differences in diet and genetic susceptibility.

The study suggested that age was the factor associated with high TG and low HDL-C at follow-up. Gender was risk factor for high TC, low HDL-C and high LDL-C at follow-up. Besides, smoking and diet control were risk factors for low HDL-C and high LDL-C at follow-up respectively. Previous studies reported that age appeared to influence dyslipidaemia risk [7, 22, 23]. Similar results were found in this study. The reason for this may be poor compliance with long-term drug treatment, more comorbidities, high economic pressure, failure to buy drugs and difficulty in making lifestyle changes among elderly patients. The mechanism of the effect of age on lipids is not well known. Other research has observed an association between dyslipidaemia and gender [7, 22, 23]. This study also showed that gender was related to high TC, low HDL-C and high LDL-C at follow-up. This may be associated with lifestyle and dietary differences.

A previous study revealed associations between dyslipidaemia and smoking [24]. The results demonstrated that smoking was significantly associated with the risk of low HDL-C at follow-up. Nicotine, caffeine and other substances in tobacco can stimulate blood vessels and cause structural changes, which leads to dyslipidaemia. Moreover, the importance of diet control is evident for the control of blood lipid levels. Besides, due to the 100% or 99% would be positive, the daily staple food intake was not be included in the regression analysis when analyzing the LDL-C.

### Study strengths and limitations

The major strength of the study was the inclusion of PCI patients, which provided a representativeness of the findings in PCI management. Standardized survey instruments and a trained interviewer guaranteed the reliability of the analyses. Some limitations of the study deserve to be mentioned. First, the patients included in 2015 and 2016 did not have a detailed follow-up plan, thus follow-up was performed only 1 year after discharge. Second, during the telephone follow-up period, the patients were only verbally asked if their lifestyle and habits had improved; therefore, there was a lack of data registration for changes in lifestyle at follow-up. Third, due to the lack of information on lifestyle changes, it was impossible to discuss the relationship between lipid profiles and lifestyle changes at follow-up. Fourth, although lipid-lowering medication data existed, the related lifestyle changes were not registered, thus the relationship between lipid profiles at follow-up and lipid-lowering medication could not analysed. Lastly, because of missing information, the comparison between participants who were followed up and participants who were not followed up were not analysed.

## Conclusions

In conclusion, after 1 year of follow-up, the mean values of lipids and the prevalence of dyslipidaemia in patients with PCI at follow-up included in the study were significantly lower than those at baseline. Gender, age, smoking, and diet control affected patients’ blood lipids. Therefore, postoperative management of discharged PCI patients is crucial and provides a basis for primary health care. However, a large sample size and long-term follow-up are needed to further explore the associations of lifestyle changes and medication with CHD, lipid profiles and end events.

## Data Availability

The datasets used or analysed during the current study are available as supplementary Tables.

## Abbreviations

CHD: Coronary heart disease
PCI: Percutaneous coronary intervention
TC: Total cholesterol
TG: Triglyceride
LDL-C: Low-density lipoprotein cholesterol
HDL-C: High-density lipoprotein cholesterol
CCU: Cardiac intensive care unit
NCEP: National Cholesterol Education Program
NCEP-ATP III: National Cholesterol Education Program (Adult Treatment Panel III)
DM: Diabetes mellitus
FPG: Fasting plasma glucose
2hPG: 2 h postprandial plasma
SBP: Systolic blood pressure
DBP: Diastolic blood pressure
BMI: Body mass index
OR: Odds ratios
